# Footedness Is Associated with Self-reported Sporting Performance and Motor Abilities in the General Population

**DOI:** 10.3389/fpsyg.2016.01199

**Published:** 2016-08-10

**Authors:** Ulrich S. Tran, Martin Voracek

**Affiliations:** Department of Basic Psychological Research and Research Methods, School of Psychology, University of ViennaVienna, Austria

**Keywords:** footedness, sporting performance, latent variable analysis, negative frequency-dependent selection hypothesis, innate superiority hypothesis

## Abstract

Left-handers may have strategic advantages over right-handers in interactive sports and innate superior abilities that are beneficial for sports. Previous studies relied on differing criteria for handedness classification and mostly did not investigate mixed preferences and footedness. Footedness appears to be less influenced by external and societal factors than handedness. Utilizing latent class analysis and structural equation modeling, we investigated in a series of studies (total *N* > 15300) associations of handedness and footedness with self-reported sporting performance and motor abilities in the general population. Using a discovery and a replication sample (*n*s = 7658 and 5062), Study 1 revealed replicable beneficial effects of mixed-footedness and left-footedness in team sports, martial arts and fencing, dancing, skiing, and swimming. Study 2 (*n* = 2592) showed that footedness for unskilled bipedal movement tasks, but not for skilled unipedal tasks, was beneficial for sporting performance. Mixed- and left-footedness had effects on motor abilities that were consistent with published results on better brain interhemispheric communication, but also akin to testosterone-induced effects regarding flexibility, strength, and endurance. Laterality effects were only small. Possible neural and hormonal bases of observed effects need to be examined in future studies.

## Introduction

Hand preference is a long studied topic in sports science, suggesting that left-handers may have an advantage over right-handers in various interactive sports, i.e., sports that involve the direct confrontation with opponents (e.g., Grouios et al., 2000, 2002a). In martial arts and boxing, there is evidence suggesting a beneficial effect of the ‘southpaw’ stance, i.e., the right-hand-and-right-foot-forward stance of the left-handed fighter, compared to the ‘orthodox’ (mirror-image) stance of the right-handed fighter (Ziyagil et al., 2010; Groothuis et al., 2014; Loffing and Hagemann, 2015). Similarly, there is evidence of an advantage of left-handedness in fencing (Harris, 2010).

While there are further accounts of left-handedness being advantageous in, for example, tennis (Loffing et al., 2012a; Breznik, 2013), basketball (Lawler and Lawler, 2011), or cricket (Brooks et al., 2004), there are also accounts that left-handers are only overrepresented in the expert domains of some sports, but are at the same time not more successful than right-handers (e.g., in combative sports; Baker and Schorer, 2013; Pollett et al., 2013). Left-handers are two to five times more frequent among top ranking players and Grand Slam finalists in tennis than among the overall population (Holtzen, 2000). However, the advantage of being left-handed appears to be declining in some professional sports in recent years (e.g., in tennis; Loffing et al., 2012a; Breznik, 2013; but see Loffing and Hagemann, 2015, for evidence of an increase of left-handed boxing).

Two major strands of argumentation are discussed in the literature as to the possible causes of a left-hand advantage in sports: First, the negative frequency-dependent selection hypothesis (FDSH; Faurie and Raymond, 2005; see also Wood and Aggleton, 1989, and references provided therein), and, second, advantageous predispositions innate to left-handers (innate superiority hypothesis). According to the FDSH, left-handers gain an advantage in interactive sports, because of the limited experience and familiarity of right-handed players with left-handed opponents (only around 10% of the general population are left-handed; e.g., Coren, 1993). The FDSH may explain both an overrepresentation of left-handers in some sports, but also the observed temporal decline regarding a left-handed advantage in professional sports (e.g., Loffing et al., 2012a; Breznik, 2013): Professional players may have recognized this advantage and intensified training specifically with regard to left-handed opponents. However, left-handers may not only have a strategic advantage over right-handers. There is also evidence that both right- and left-handers are less apt to anticipate and perceive movement patterns of left-handers, because the visual system is less accustomed to left-handed than right-handed movements (negative perceptual frequency effect hypothesis; e.g., Hagemann, 2009; Loffing et al., 2012b; see also Marzoli et al., 2015); however, this effect may be diminished by training (e.g., Schorer et al., 2012).

As to possible innate superior abilities, left-handers may have less brain hemispheric lateralized motor control than right-handers, which may benefit bimanual coordination and hand skill (e.g., Gorynia and Egenter, 2000; Nalçacı et al., 2001; Buckingham et al., 2011). Moreover, there is evidence of a higher number or density of axons in the corpus callosum in left-handers (Westerhausen et al., 2004), which may benefit the speed of the integration of sensory and motor information across the brain hemispheres (Cherbuin and Brinkman, 2006), and which possibly also allows for reported faster and more accurate movements in sport among left-handers, compared to right-handers (Dane and Erzurumluoglu, 2003).

Recent evidence (Tran et al., 2014a) points out that psychometrically assessed lateral preferences for hand, foot, eye, and ear are discrete and trichotomous, i.e., comprise right, mixed, and left preferences. Moreover, preferences may be further explained by underlying sidedness, which, similarly, contains a mixed class, and whose most important predictor is footedness (Tran et al., 2014a). Distinguishing only left from right preferences (e.g., Wood and Aggleton, 1989; Loffing et al., 2012a), previous research often did not take into account mixed preferences (but see, e.g., Carey et al., 2001). Criteria and cutoffs utilized for the classification of handedness were often arbitrary. Footedness was mostly not investigated (but see, e.g., Carey et al., 2001; Grouios et al., 2002b), even though it may be a better indicator of brain lateralization (Elias and Bryden, 1998), being less influenced by external and societal factors than handedness (Porac and Coren, 1981; Tran et al., 2014a). Mixed-handedness is uncommon in the population (2–7%), but mixed-footedness is much more common (26–35%; Tran et al., 2014a).

Here, we present the results of two studies. Study 1 is a large-scale study, investigating the associations of handedness and footedness with self-reported sporting performance in two large and independent general population samples. We used short measures of lateral preferences, but latent variable modeling for classification (i.e., latent class analysis, LCA), assuring high reliability of classification. Investigating primarily everyday sportspersons and not selecting for practiced type of sport allowed examining advantages of non-right-handedness (and non-right-footedness) across various sports and their real-life impact. We tested whether laterality effects generalized across different types of sport, and measured their magnitude among mostly not professionally trained sportspersons of the general population. By using a discovery-replication-sample design we also tested the replicability and the robustness of observed effects.

Study 2 investigated the associations of handedness and footedness with sporting performance in a further large general population sample, utilizing more comprehensive measures of lateral preferences and again latent variable analysis. We examined whether footedness may be a two-factor, rather than a one-factor, construct (e.g., Kalaycıoğlu et al., 2008), and whether different factors of footedness were differentially associated with sporting performance. Moreover, associations of handedness and footedness with self-reported motor abilities were examined to gain an insight into possible innate abilities of non-right-handers (and non-right-footers) that may be generally beneficial in sport on the amateur level. As previous research suggested a moderating effect of sex on laterality effects in some sports (e.g., Dane and Erzurumluoglu, 2003; Harris, 2010; Breznik, 2013), sex effects were investigated and controlled for in both studies.

## Study 1

Study 1 used a discovery-replication-sample design (McCarthy et al., 2008), following recent recommendations to counteract potentially false-positive and thus irreproducible research findings (Asendorpf et al., 2013). Independent discovery and replication samples within the same study are also considered best practice in genome-wide association studies, in order to guard against false-positive findings and to demonstrate the robustness of an effect, if the replication is successful (McCarthy et al., 2008).

### Methods

#### Participants

Two large and independent discovery (*n* = 7658) and replication (*n* = 5062) samples were used for this research (**Table 1**). Samples included participants from the general population with a broad age range and slightly more women than men. The majority of the participants were Austrian or German, other nationalities included Italian (1.4%), Turkish (0.6%), and Romanian (0.4%). Findings reported here are part of a larger study of which part of the data has already been published elsewhere (Tran et al., 2014a,b).

**Table 1:** Sample characteristics in Studies 1 and 2.

	Study 1		
	Discovery sample	Replication sample	Study 2
*N*	7658	5062	2592
Women, *n* (%)	4456 (58.2%)	2749 (54.3%)	1394 (53.8%)
Age, range (years)	18–89	18–92	18–93^a^
Interquartile range	22–35	22–48	23–48^a^
Mean (*SD*)	30.24 (12.62)	35.74 (16.05)	35.29 (15.47)^a^
Nationality, *n* (%)^b^			
Austria	5339 (70.0%)	3415 (67.7%)	1579 (61.1%)
Germany	1773 (23.2%)	1310 (26.0%)	771 (29.8%)
Other	514 (6.7%)	316 (6.3%)	233 (9.0%)

*Analysis *n*s = ^a^2567, ^b^7626/5041/2583.*

#### Materials

Handedness and footedness were assessed with four items each out of the Lateral Preference Inventory (LPI; Coren, 1993). Items asked for (a) hand preference with regard to: Writing, throwing a ball to hit a target, using a knife to cut something without simultaneously using a fork, and using a hammer to drive a nail into something; (b) foot preference with regard to: Kicking a ball to hit a target, picking up a pebble with the toes, stepping on a beetle or a cigarette stump, and stepping up onto a chair. Handedness items had been selected *a priori* with respect to broad trait coverage, item performance indicators, and balance of fine- vs. gross motor skills. Response options were *left, either*, and *right* (in this order), coded -1, 0, and +1, respectively. Scales of handedness and footedness have been shown to be unidimensional in previous research with the current two samples (Tran et al., 2014a). Cronbach α for handedness were 0.91 (discovery sample) and 0.92 (replication sample), and for footedness 0.71 and 0.73, respectively.

Level of sporting performance was assessed with one item, asking respondents to rank their sporting achievement on a 10-point scale^1^ (Manning and Pickup, 1998). Scale scores have clear content and face validity; rankings in this scale have been further shown to correlate with best 800 and 1500 m times among middle distance runners (Manning and Pickup, 1998).

#### Procedure

Data were collected by a large number of data collectors in two waves, separated by about half a year, in the course of a larger project on individual difference variables. Waves of data collection were independent from each other with regard to the data collectors and participants involved. Participants were approached on a personal basis, using word-of-mouth and personal contacts. Participants had to be fluent in German, which was the survey language. There were no exclusion criteria, apart from insufficient language proficiency. Study participation was voluntary, anonymous, and participants were not remunerated for participation. This study was carried out in accordance with all relevant requirements and recommendations of national (Austrian) and EU law and the Declaration of Helsinki; all subjects provided informed consent.

#### Analysis

Handedness and footedness were classified independently in the two samples with LCA (Collins and Lanza, 2010). Similar to factor analysis, LCA explains associations between observed variables by introducing latent, not directly observable, variables. However, in contrast to factor analysis, the latent variable in LCA is not a continuum. Instead, it consists of a number of discrete classes. Associations between observed variables are explained conditional on class membership in LCA. LCA has been frequently used for the classification of handedness before and has been shown to be an adequate method for classifying subjects with respect to their ratings in lateral preference inventories (e.g., Dragovic and Hammond, 2007). The fit of models with increasing numbers of latent classes was examined, looking for the smallest number of classes that explained the data best. For analysis, Latent GOLD 4.5 was utilized. Model fit was assessed with the frequently used Bayesian information criterion (BIC), percentages of classification error, and the likelihood-ratio goodness of fit statistic (*L*^2^). Lower BIC values, lower percentages of classification error, and lower and preferably not significant *L*^2^ values are indicative of a better model fit when comparing competing models. Obtained latent classes of the final models were used for further analysis.

Generalized linear models (GLM) were utilized to examine associations of handedness and footedness with level of sporting performance, contrasting classes with non-right preferences against right preference classes, using dummy coding. As level of sporting performance scores was scored on an ordered-categorical scale, ordinal logistic regression analysis was performed in GLM analysis. The dependent variable was modeled with a multinomial distribution, using as a link function the cumulative logistic function. Analysis controlled for age, sex, and sample, and tested the replicability of findings by including an interaction term with sample. In order to maximize statistical power with regard to possible sex differences, interactions with sex were further investigated by including interaction terms in the model, but independent analyses for men and women were also run.

### Results and Discussion

#### Handedness and Footedness

Latent class analysis suggested three classes of handedness and footedness each (**Table 2**; results reported here have been previously published in Tran et al., 2014b). LCA computes for each subject the probability of belonging to each of the latent classes. Based on the highest probability, subjects are assigned to latent classes. Posterior assignment probabilities (i.e., the mean probability of belonging to the assigned class) were high (handedness: 89–99% for the three classes in the two samples; footedness: 89–93%), indicating a high reliability of classification. **Table 3** displays univariate and joint distributions of handedness and footedness, combining both samples. In majority, participants were right-handed (89.6%) and right-footed (61.6%); mixed-handedness was less frequent than left-handedness (2.3% vs. 8.1%), whereas mixed-footedness was more frequent than left-footedness (30.2% vs. 8.2%). Handedness and footedness were concordantly associated [χ^2^(4) = 4219.24, *p* < 0.001]: Compared to non-right-handers, right-handers were more likely right-footed than mixed-footed (*OR* = 5.02, 95% confidence interval = [4.24–5.94], *p* < 0.001) and more likely right-footed than left-footed (*OR* = 59.35 [49.30–71.45], *p* < 0.001); compared to right-handers, left-handers were more likely left-footed than right-footed (*OR* = 65.51 [53.87–79.67], *p* < 0.001) and more likely left-footed than mixed-footed (*OR* = 20.76 [17.32–24.88], *p* < 0.001); mixed-handers were more likely mixed-footed or left-footed than right-footed (*OR* = 16.19 [11.25–23.30], *p* < 0.001; all comparisons based on Bonferroni-corrected contrast tests, overall *p* < 0.05).

**Table 2:** Fit of the latent class models in the discovery and replication samples (Study 1).

Model	BIC	*L*^2^	*df*	*p*	Classification error, %
**Handedness**
1-Cluster	23402.59	8441.22	184	<0.001	–
	15373.05	5871.32	176	<0.001	–
2-Cluster	15696.66	927.79	175	<0.001	0.52
	10122.31	543.80	167	<0.001	0.49
3-Cluster	15335.10	212.74	166	0.008	1.03
	9833.65	178.38	158	0.130	1.05
4-Cluster	15336.69	133.84	157	0.910	3.82
	9864.01	131.98	149	0.840	3.58
**Footedness**
1-Cluster	49956.20	6806.28	192	<0.001	–
	32566.53	4877.79	200	<0.001	–
2-Cluster	45657.88	2427.47	183	<0.001	8.34
	29602.76	1837.25	191	<0.001	8.46
3-Cluster	43708.27	397.36	174	<0.001	9.63
	28221.43	379.16	182	<0.001	9.02
4-Cluster	43695.42	304.02	165	<0.001	15.77
	28203.46	284.42	173	<0.001	12.81

*BIC, Bayes information criterion; *L*^2^, likelihood ratio test statistic. Cell entries list the fit of alternative 1-, 2-, 3- and 4-cluster models in the discovery sample (first line) and the replication sample (second line) with regard to three indices of model fit. Lower values of BIC, *L*^2^, and classification error indicate a better model fit.*

**Table 3:** Univariate and joint distributions of handedness and footedness in the combined discovery and replication samples (Study 1).

	Right-footed	Mixed-footed	Left-footed	Total
Right-handed	7628 (60.0%)	3373 (26.5%)	396 (3.1%)	11397 (89.6%)
Mixed-handed	33 (0.3%)	219 (1.7%)	45 (0.4%)	297 (2.3%)
Left-handed	177 (1.4%)	247 (1.9%)	602 (4.7%)	1026 (8.1%)
Total	7838 (61.6%)	3839 (30.2%)	1043 (8.2%)	

Controlling for sex, age, sample, and handedness in multinomial regression analysis, younger participants were more likely to be mixed-footed (*OR* = 0.98, 95% confidence interval = [0.98–0.99], *p* < 0.001) than older participants. Moreover, men were more likely to be mixed-footed (*OR* = 1.30 [1.20–1.41], *p* < 0.001) or left-footed (*OR* = 1.30 [1.11–1.52], *p* = 0.001) than women.

#### Associations with Sporting Performance

Descriptive statistics on level of sporting performance and practiced types of sport are presented in **Table 4**. Level of sporting performance was higher in younger participants and men reported higher ranks than women (**Table 5**). Controlling for these effects, mixed-footers were overall more likely to report higher ranks than all others. There were no interactions of handedness or footedness with sex (*p*s ≥ 0.125), and the effect of mixed-footedness was also replicable across the two samples (interaction of mixed-footedness with sample: *OR* = 0.93 [0.81–1.07], *p* = 0.322). The effect of mixed-footedness was further also present in independent analyses of men (*OR* = 1.31 [1.18–1.46], *p* < 0.001) and women (*OR* = 1.18 [1.07–1.31], *p* = 0.001).

**Table 4:** Level of sporting performance and practiced sports in Studies 1 and 2, and motor skills in Study 2.

	Study 1		
	Discovery sample	Replication sample	Study 2
Level of sporting performance, range^a^	1–10	1–10	1–10
Interquartile range	2–4	2–4	2–4
Mean (SD)	3.13 (2.14)	3.07 (2.17)	3.25 (2.01)
Practiced sports, *n* (%)^b^			
Interactive team sports^c^	1268 (16.9%)	738 (15.0%)	432 (16.9%)
Running	851 (11.4%)	577 (11.7%)	315 (12.3%)
Gymnastics	584 (7.8%)	353 (7.2%)	186 (7.3%)
Racket sports^d^	474 (6.3%)	314 (6.4%)	172 (6.7%)
Cycling	301 (4.0%)	231 (4.7%)	179 (7.0%)
Swimming	301 (4.0%)	177 (3.6%)	134 (5.2%)
Dancing^e^	282 (3.8%)	149 (3.0%)	68 (2.7%)
Martial arts and fencing	271 (3.6%)	155 (3.2%)	82 (3.2%)
Skiing	250 (3.3%)	134 (2.7%)	96 (3.8%)
Athletics	136 (1.8%)	79 (1.6%)	57 (2.2%)
Other type of sport^f^	665 (8.9%)	397 (8.1%)	244 (9.5%)
Not indicated	729 (9.7%)	606 (12.3%)	257 (10.1%)
No sport	1376 (18.4%)	1010 (20.5%)	334 (13.1%)
Motor skills, mean (*SD*)^g^			
Flexibility	–	–	2.80 (0.72)
Coordination	–	–	2.85 (0.60)
Strength	–	–	2.65 (0.77)
Speed	–	–	2.69 (0.70)
Endurance	–	–	2.48 (0.85)

*Analysis *n*s = ^a^7486/4917/2521, ^b^7488/4920/2556, ^g^2591. ^c^Including soccer,volleyball, basketball, handball, rugby, hockey, and American football; ^d^including tennis, squash, badminton, and table tennis; ^e^competition ballroom dancing and other forms of dancing; ^f^non-interactive sport not listed above (e.g., yoga or horse-riding).*

**Table 5:** Predictors of level of sporting performance (Study 1).

	Odds Ratio (95% confidence interval)
Mixed-handed	0.77 [0.53–1.11]
Left-handed	0.97 [0.80–1.18]
Mixed-footed	**1.38 [1.20–1.58]^∗∗∗^**
Left-footed	1.19 [0.96–1.47]
Control variables : Sex (male)	**2.05 [1.20–3.48]^∗∗^**
Age	**0.97 [0.97–0.98]^∗∗∗^**
Sample	1.06 [0.94–1.20]

*Analysis *n* = 12403. Right preferences served as reference categories for effect tests of handedness and footedness. Significant effects (*p* < 0.05) are printed boldface. The model included also interaction terms for lateral preferences with sex, and for mixed-footedness with sample, which were not significant and are not shown for brevity (see Section “Associations with Sporting Performance” of Study 1). ^∗∗^*p* < 0.01, ^∗∗∗^*p* < 0.001.*

Controlling for sex, age, sample, and modalities of handedness and footedness, a significant positive effect of mixed-footedness on level of sporting performance could be individually replicated for team sports (*n* = 2006; *OR* = 1.46 [1.23–1.74], *p* < 0.001), dancing (*n* = 431; *OR* = 1.59 [1.11–2.27], *p* = 0.011), and swimming (*n* = 478; *OR* = 1.50 [1.01–2.25], *p* = 0.045). The effect was further of equal magnitude for skiing (*n* = 384; *OR* = 1.48 [0.96–2.27], *p* = 0.075), albeit nominally missing significance. Further, there were significant positive effects of left-footedness for team sports (*n* = 2006; *OR* = 1.44 [1.05–1.99], *p* = 0.026) and martial arts and fencing (*n* = 426; *OR* = 2.06 [1.04–4.07], *p* = 0.037).

Study 1 provided replicable evidence of specific effects of mixed-footedness and, to a lesser extent, of left-footedness on sporting performance in various interactive, but also non-interactive, sports. Whereas previous research focused mostly on handedness, footedness, and, specifically, mixed-footedness, proved the most important predictor of sporting performance in Study 1, utilizing large general population samples that comprised mostly everyday sportspersons. Thus, results corroborated a greater importance of footedness over handedness in laterality research (Tran et al., 2014a).

Observed effects of non-right lateral preferences, i.e., left-footedness, in team and combat sports appear consistent with strategic and negative perceptual frequency effects. However, mixed-footedness was beneficial in both interactive (team sports) and non-interactive sports (dancing, swimming, and skiing), suggesting that it may be a specific indicator of innate superior abilities. Recent evidence shows that mixed, rather than left, lateral preferences may be associated with better brain interhemispheric communication (Davidson and Tremblay, 2013). Complementing these findings on a behavioral level, our results indicate that mixed-footedness may consistently benefit the performance in a larger range of sports with a small effect size.

## Study 2

There is evidence that footedness may consist of two factors, differentiating skilled and unskilled/movement tasks (e.g., Kalaycıoğlu et al., 2008; Schneiders et al., 2010). Further, in Study 1 no conclusions could be drawn on which motor abilities lateral preferences might have an effect. Therefore, Study 2 was performed, utilizing more items to assess handedness and footedness, and assessing simultaneously self-reported motor abilities in a large general population sample, besides level of sporting performance. We expected to replicate effects of mixed-footedness of Study 1, but were interested in whether footedness was still a unidimensional construct, utilizing a larger item bank for its assessment.

### Methods

#### Participants

A novel sample with a broad age range was again drawn from the general population (**Table 1**). As in Study 1, there were slightly more women than men. Again, the majority of the participants were Austrian or German. Findings reported here are part of a larger study of which part of the data has already been published elsewhere (Tran and Voracek, 2015).

#### Materials

Handedness was assessed with a 10-item scale (Tran et al., 2014a). This scale comprised the four items of Study 1, but also six further items of the LPI and of the Edinburgh Handedness Inventory (EHI; Oldfield, 1971). Together, these 10 items were found to be the most reliable indicators of handedness in independent data in previous research (Tran et al., 2014a). Response options were *always right, usually right, no preference, usually left*, and *always left* (in this order), coded +2, +1, 0, -1, and -2, respectively. Response options *always* and *usually* were combined for analysis, following previous recommendations (Tran et al., 2014a). Cronbach α was 0.97 in the current sample.

Footedness was assessed with a 9-item scale that included the four items of Study 1, but also five more items taken from Kalaycıoğlu et al. (2008), asking for foot preference with regard to: First foot when stepping forward, when stepping up stairs, and when stepping up stairs backward; tracing a letter while standing, and erasing that letter. These items, excluding ‘tracing a letter,’ loaded highest on a factor of unskilled bipedal movement tasks in Kalaycıoğlu et al. (2008) and were therefore selected for the current study; ‘tracing a letter’ loaded, along with the four items already included in the scale, highest on a factor of skilled unipedal tasks. Items were scored as for handedness. Cronbach α of the 9-item scale was 0.89 in the current sample.

Motor abilities were assessed with five scales (Stiller et al., 2004), measuring flexibility, coordination, strength, speed, and endurance with six items each (five for speed). The scales have been validated in large samples of children, adolescents, and young adults, and have shown good reliability and good factorial, discriminant, and construct validity (Stiller et al., 2004). Items were scored on 4-point scales, asking for the degree of agreement with various statements on physical abilities. Cronbach α in the current sample was (above order) 0.87, 0.86, 0.90, 0.81, and 0.91.

#### Procedure

The procedure was similar to Study 1, see Section “Procedure.”

#### Analysis

Handedness and footedness were again classified with LCA as in Study 1, see Section “Analysis.” Prior to classification, the dimensionality of the nine footedness items was investigated with exploratory structural equation modeling (ESEM; Asparouhov and Muthén, 2009). ESEM integrates exploratory and confirmatory approaches and allows for a flexible examination of factor structures, in that it freely measures cross-loadings as in exploratory factor analysis. At the same time, ESEM provides standard errors and goodness-of-fit statistics as in conventional SEM. Previous research provided evidence for non-negligible cross-loadings of footedness items in multidimensional models (e.g., Kalaycıoğlu et al., 2008; Schneiders et al., 2010), treating items as continuous indicators, however. Response distributions of items in lateral preference inventories are typically highly skewed. Factoring such items may result in an overextraction of factors (Bernstein and Teng, 1989). In contrast to previous studies, items were thus treated as ordered-categorical in the present study, utilizing the weighted least square mean- and variance-adjusted (WLSMV) estimator of Mplus 6.11 that is based on the polychoric correlation matrix of the items. We fitted a 2-factor ESEM model to the data, assessing model fit with the comparative fit index (CFI), the Tucker-Lewis index (TLI), and the root mean square error of approximation (RMSEA), using benchmarks of Hu and Bentler (1999) (CFI/TLI: good fit: ≥ 0.95, acceptable fit: ≥0.90; RMSEA: good fit: <0.06, acceptable fit: <0.08). As in models with small degrees of freedom RMSEA values may be inflated (Kenny et al., 2014), primarily the CFI and TLI were utilized to evaluate model fit.

Generalized linear models were again utilized for examining effects of handedness and footedness, controlling for age and sex. In analyses of motor abilities a normal distribution was used to model the dependent variables and the identity function was utilized as the link function. Again, possible sex differences were investigated by including interaction terms with sex in the models, but also by conducting independent analyses in samples of men and women. Analysis of level of sporting performance proceeded as in Study 1, see Section “Analysis.”

### Results and Discussion

#### Dimensionality of Footedness

A unidimensional model fitted unsatisfactorily on the data [χ^2^(27) = 1383.43, *p* < 0.001, CFI = 0.922, TLI = 0.896, RMSEA = 0.142, 90% confidence Interval = [0.136–0.148]; results reported here have been previously published in Tran and Voracek, 2015]. In contrast, a two-dimensional model provided a much better fit [χ^2^(13) = 304.32, *p* < 0.001, CFI = 0.982, TLI = 0.962, RMSEA = 0.095 [0.086–0.104]]. ‘Stepping up stairs backward’ had to be excluded, however, as it loaded only weakly on the two factors (0.26 and 0.26). The loading pattern of the 8-item scale is presented in **Table 6**. Five items loaded highly on factor 1, subsuming skilled unipedal tasks (denominated skilled-footedness from here on) three items loaded highly on factor 2, subsuming unskilled bipedal movement tasks (movement-footedness). Our use of the term ‘skilled-footedness’ here is in agreement with the use of this term by other authors (e.g., Kalaycıoğlu et al., 2008; Schneiders et al., 2010). All tasks that loaded on the second factor comprised bipedal changes in position, i.e., movements. Hence, we decided to denominate this factor for what it is, a factor of ‘movement-footedness,’ rather than utilizing the less specific term ‘unskilled footedness’ that is more commonly used by other authors (e.g., Kalaycıoğlu et al., 2008; Schneiders et al., 2010).

**Table 6:** Standardized factor loadings of eight footedness items in the two-factor ESEM analysis (Study 2).

Item	Skilled	Movement
Kicking a ball	**0.67**	0.24
Picking up a pebble with toes	**0.72**	0.15
Stepping on a beetle/cigarette	**0.65**	0.21
Stepping up onto a chair	0.17	**0.72**
Stepping forward	-0.02	**0.91**
Stepping up stairs	0.01	**0.90**
Trace a letter while standing	**0.92**	-0.04
Erase the letter	**0.81**	-0.01

*Boldface marks the factor where items loaded highest. All loadings were significant (*p*s < 0.001), except for loadings that were ≤| 0.04 | (*p*s ≥ 0.078).*

The factors inter-correlated with *r* = 0.58 (*p* < 0.001). Cronbach α of the 5-item scale was 0.86 and of the 3-item scale 0.87. Our results corroborate thus that footedness, other than handedness (Tran et al., 2014a), is a two-dimensional construct.

#### Handedness and Footedness

Latent class analysis suggested three classes of handedness and footedness, in both modalities, each (**Table 7**; results reported here have been previously published in Tran and Voracek, 2015); allowing for correlated residuals of items ‘trace a letter while standing’ and ‘erasing the letter’ improved model fit further in skilled-footedness. Posterior assignment probabilities of participants to classes were again high (handedness: 88–99%; skilled-footedness: 86–95%; movement-footedness: 94–97%). **Table 8** displays univariate and joint distributions of handedness and the two modalities of footedness. Using the 10-item scale, there were more mixed-handers in the current sample than in Study 1 (7.2% vs. 2.3%); however, the current figure closely matched a previous estimate with that scale in independent data (7.1%; Tran et al., 2014a). Proportions of left-footers (skilled: 10.2%; movement: 13.7%) were higher than of left-footers in Study 1 (8.2%), utilizing a 4-item scale not discriminating for skilled and unskilled tasks. In turn, modalities of mixed-footedness (skilled: 23.7%; movement: 23.9%) were each less frequent than mixed-footedness in Study 1 (30.2%).

**Table 7:** Fit of the latent class models (Study 2).

Model	BIC	*L*^2^	*df*	*p*	Classification error, %
**Handedness**					
1-Cluster	21122.78	12411.38	2572	<0.001	–
2-Cluster	11643.70	2767.23	2551	0.002	0.22
3-Cluster	10903.68	1862.15	2530	1.000	1.94
4-Cluster	10811.76	1605.17	2509	1.000	2.03
**Skilled-footedness**					
1-Cluster	18808.55	4070.62	726	<0.001	–
2-Cluster	16342.86	1518.47	715	<0.001	4.35
3-Cluster	15634.84	723.99	704	0.293	7.97
	15355.58^a^	413.29ˆa	700^a^	1.000ˆa	8.34ˆa
4-Cluster	15560.79	563.48	693	1.000	10.76
**Movement-footedness**					
1-Cluster	14153.93	3061.59	48	<0.001	–
2-Cluster	12078.54	931.17	41	<0.001	4.48
3-Cluster	11247.37	44.97	34	0.098	5.54
4-Cluster	11299.70	42.29	27	0.031	12.73

*BIC, Bayes information criterion; L^2^, likelihood ratio test statistic. ^a^Allowing for correlated residuals of items ‘trace a letter while standing’ and ‘erasing the letter.’ Cell entries list the fit of alternative 1-, 2-, 3-, and 4-cluster models in the total sample with regard to three indices of model fit. Lower values of BIC, *L*^2^, and classification error indicate a better model fit.*

**Table 8:** Univariate and joint distributions of handedness and of two modalities of footedness (Study 2).

	Footedness						
	Skilled			Movement			
	Right-footed	Mixed-footed	Left-footed	Right-footed	Mixed-footed	Left-footed	Total
**Handedness**							
Right-handed	1627 (62.8%)	486 (18.8%)	65 (2.5%)	1495 (57.7%)	490 (18.9%)	193 (7.4%)	2178 (84.0%)
Mixed-handed	59 (2.3%)	90 (3.5%)	38 (1.5%)	80 (3.1%)	69 (2.7%)	38 (1.5%)	187 (7.2%)
Left-handed	26 (1.0%)	39 (1.5%)	162 (6.3%)	44 (1.7%)	60 (2.3%)	123 (4.7%)	227 (8.8%)
**Skilled-Footedness**							
Right-footed				1302 (50.2%)	266 (10.3%)	144 (5.6%)	1712 (66.0%)
Mixed-footed				262 (10.1%)	295 (11.4%)	58 (2.2%)	615 (23.7%)
Left-footed				55 (2.1%)	58 (2.2%)	152 (5.9%)	265 (10.2%)
Total	1712 (66.0%)	615 (23.7%)	265 (10.2%)	1619 (62.5%)	619 (23.9%)	354 (13.7%)	

Handedness and skilled-footedness were concordantly associated [χ^2^(4) = 1174.01, *p* < 0.001]: With respect to overall marginal proportions, right-handers were more likely to be right- than left-footed, left-handers more likely left- than right-footed, and mixed-handers were more likely to be mixed- or left-footed than right-footed (all *p*s < 0.05, Bonferroni-corrected). Even though somewhat weaker [χ^2^(4) = 423.75, *p* < 0.001], handedness and movement-footedness were similarly concordantly associated, as were the two modalities of footedness with one another [χ^2^(4) = 776.11, *p* < 0.001].

Controlling for sex, age, and both modalities of footedness in multinomial regression analysis, younger participants were more likely to be mixed-handed than older participants (*OR* = 0.99, 95% confidence interval = [0.97–1.00], *p* = 0.021). Controlling for handedness and movement-footedness, a similar age effect emerged for mixed preferences in skilled-footedness (*OR* = 0.98 [0.98–0.99], *p* < 0.001) and left preferences in skilled-footedness (*OR* = 0.98 [0.97–0.99], *p* = 0.007). Vice versa, controlling for handedness and skilled-footedness, younger participants were also more likely to report mixed preferences in movement-footedness (*OR* = 0.98 [0.98–0.99], *p* < 0.001). Men were more likely to report left preferences in skilled-footedness (*OR* = 1.47 [1.01–2.14], *p* = 0.043) and movement-footedness (*OR* = 2.82 [2.15–3.70], *p* < 0.001), and more likely to report mixed preferences in movement-footedness (*OR* = 1.75 [1.43–2.14], *p* < 0.001) than women.

#### Associations with Motor Abilities

Descriptive statistics on motor abilities are presented in **Table 4**. **Table 9** lists the results of the Wald effect tests in the GLMs. With regard to motor abilities, flexibility (*b* = -0.006, 95% confidence interval = [-0.008 to -0.001]), coordination (*b* = -0.004 [-0.003 to -0.005]), speed (*b* = -0.008 [-0.010 to -0.006]), and endurance (*b* = -0.010 [-0.012 to -0.008]) were all higher in younger participants. Men reported higher scores than women in coordination (*d* = 0.13 [0.05–0.21]), strength (*d* = 0.19 [0.11–0.27]), speed (*d* = 0.21 [0.13–0.29]), and endurance (*d* = 0.20 [0.12–0.28]). Controlling for these effects, left-handedness had a negative effect on coordination (*d* = -0.14 [-0.27 to -0.002]) and speed (*d* = -0.18 [-0.32 to -0.04]). In contrast, mixed preferences in skilled-footedness had a positive effect on flexibility (*d* = 0.10 [0.01–0.20]), coordination (*d* = 0.10 [0.01–0.19]), and speed (*d* = 0.12 [0.02–0.21), whereas in movement-footedness specifically on coordination (*d* = 0.12 [0.03–0.21]). Among women, mixed preferences in skilled-footedness had a positive effect on endurance [*d* = 0.20 [0.08–0.33], *p* = 0.002; no effect among men, *p* = 0.932; test of the interaction: χ^2^(1) = 4.98, *p* = 0.026]. Left preferences in skilled-footedness among both men and women had a positive effect on flexibility (*d* = 0.13 [0.01–0.26]). Among men, left preferences in movement-footedness had a positive effect on strength [*d* = 0.23 [0.09–0.38], *p* = 0.002; no effect among women, *p* = 0.793; test of the interaction: χ^2^(1) = 4.16, *p* = 0.041].

**Table 9:** Wald effect tests in the generalized linear models for motor abilities (Study 2).

	Flexibility	Coordination	Strength	Speed	Endurance
Mixed-handed	1.35	0.38	1.83	1.15	0.02
Left-handed	2.20	**3.94^∗^**	0.24	**6.71^∗∗^**	2.67
Mixed-footed (skilled)	**5.04^∗^**	**4.90^∗^**	0.11	**6.18^∗^**	**4.42^∗^**^,a^
Left-footed (skilled)	**4.19^∗^**	0.41	0.50	1.93	1.47
Mixed-footed (movement)	2.55	**6.87^∗∗^**	1.52	3.04	0.48
Left-footed (movement)	0.25	3.24	**2.59**^a^	2.15	0.38
Control variables: Sex (male)	0.73	**10.79^∗∗∗^**	**22.12^∗∗∗^**	**26.68^∗∗∗^**	**25.24^∗∗∗^**
Age	**48.03^∗∗∗^**	**29.59^∗∗∗^**	2.78	**79.33^∗∗∗^**	**105.79^∗∗∗^**

*Analysis *n* = 2566. Figures are Wald χ^2^ tests with one degree of freedom. Right preferences served as reference categories for effect tests of handedness and footedness. Significant effects (*p* < 0.05) are printed boldface. ^a^This effect was qualified by an interaction with sex (see Section Associations with Sporting Performance for further details); all models included interaction terms for lateral preferences and sex, which were otherwise not significant (*p*s > 0.05) and are not shown for brevity. ^∗^*p* < 0.05, ^∗∗^*p* < 0.01, ^∗∗∗^*p* < 0.001.*

The results suggest a wide range of effects of lateral preferences on motor abilities, albeit with only small effect sizes. Mostly, mixed-footedness (either for skilled or movement tasks) exerted positive effects that appear consistent (coordination, speed) with a presumably better brain interhemispheric communication (Davidson and Tremblay, 2013). The most salient effect was that of mixed preferences in movement-footedness on coordination (see χ^2^ values of effect tests in **Table 9**).

Effects of left and mixed preferences in skilled-footedness on flexibility (both sexes) and endurance (women), and of left preferences in movement-footedness on strength (men), appear further consistent with theories of intrauterine testosterone-induced effects on cerebral lateralization, promoting right-hemisphere dominance, and left-handedness (e.g., Geschwind and Galaburda, 1987; see Tran et al., 2014b, for recent replicable, albeit indirect, evidence). The evidence for an association of circulating testosterone with handedness in adults is mixed (but see Faurie et al., 2011). Testosterone reduces fat mass and increases muscle strength and leg power (Herbst and Bhasin, 2004). In women, low testosterone predicts cardiovascular events (Sievers et al., 2010).

Negative effects of left-handedness on speed were previously reported by Ziyagil (2011), directly measuring running speed in a sample of adolescent novice wrestlers. Left-handers are overrepresented among patients with developmental coordination disorder (Cairney et al., 2008; Goez and Zelnik, 2008), affecting the planning and temporal coordination of movements. This may explain negative effects observed here and elsewhere.

#### Associations with Sporting Performance

Descriptive statistics on level of sporting performance and practiced types of sport are presented in **Table 4**. **Table 10** presents predictors of level of sporting performance, independent for men and women. Level of sporting performance was among both sexes higher in younger participants. Controlling for this, left, but not mixed (*p* = 0.161), preferences for movement-footedness predicted higher sporting performance among men. Among women, mixed, but not left (*p* = 0.543), preferences for movement-footedness predicted higher sporting performance. This differential effect was not visible in a combined analysis of men and women, where left preferences in movement-footedness attained significance (*OR* = 1.79 [1.33–2.42], *p* < 0.001), but neither mixed preferences in movement-footedness (*OR* = 1.19 [0.92–1.54], *p* = 0.188) nor their interactions with sex (*p*s = 0.069 and 0.308; same order as above). Given the small magnitude of the potential differential effects among men and women, we consider the interaction tests with regard to sex as underpowered. Controlling also for motor abilities in the GLMs, effects of left-footedness among men (*OR* = 1.53 [1.12–2.09], *p* = 0.008) and mixed-footedness among women (*OR* = 1.36 [1.02–1.80], *p* = 0.026) remained intact. Thus, we interpret these results as true differential effects of footedness among men and women in the present sample. In the combined analysis, and similar to the results in Study 1, men were again more likely than women to report higher ranks of sporting performance (*OR* = 2.25 [1.01–5.02], *p* = 0.048).

**Table 10:** Predictors of level of sporting performance among men and women (Study 2).

	Men	Women
Mixed-handed	0.86 [0.56–1.31]	0.77 [0.50–1.18]
Left-handed	0.65 [0.40–1.06]	0.94 [0.56–1.56]
Mixed-footed (skilled)	1.15 [0.89–1.49]	1.12 [0.87–1.45]
Left-footed (skilled)	1.18 [0.74–1.88]	1.12 [0.62–1.96]
Mixed-footed (movement)	1.19 [0.92–1.55]	**1.43 [1.10–1.87]^∗∗∗^**
Left-footed (movement)	**1.78 [1.30–2.44]^∗∗∗^**	1.09 [0.67–1.80]
*Control variable*: Age	**0.97 [0.96–0.98]^∗∗∗^**	**0.97 [0.96–0.97]^∗∗∗^**

Analysis *n* = 1164 (men) and 1333 (women). Numbers are odds ratios with 95% confidence intervals. Right preferences served as reference categories for effect tests of handedness and footedness. Significant effects (*p* < 0.05) are printed boldface. ***p < 0.001.

Practiced sports did not deviate from Study 1, with the exception that there were slightly more participants in Study 2 practicing swimming and cycling, and less participants practicing no sport [χ^2^(11) = 101.79, *p* < 0.001; see **Table 4**]. In a supplementary analysis, the analysis sample was restricted to participants practicing team sports, martial arts and fencing, swimming, skiing, or dancing, for which Study 1 had individually uncovered effects of footedness each. Controlling for age, sample, and modalities of handedness and footedness, we observed in this subsample among men (*n* = 459) a significant positive effect of left preferences in movement-footedness (*OR* = 1.61 [1.04–2.49], *p* = 0.033) on sporting performance, and among women (*n* = 346) of left and mixed preferences (*OR* = 3.35 [1.44–7.81], *p* = 0.005; *OR* = 1.76 [1.12–2.76], *p* = 0.014). Among men, the subsample was dominated by practitioners of team sports (63.6%; all remaining categories <12% each), whereas among women, it was dominated by practitioners of team sports (39.4%) and swimming (25.1%; all remaining categories <16% each).

Summing up, movement-footedness appeared more important regarding overall sporting performance than skilled-footedness, even though the latter was found beneficial for a range of motor abilities, too. Deviating from Study 1, differential effects of foot preference among men and women emerged in Study 2. We suggest interpreting this with regard to sample size and effect size, and not as true sex differences. Effects were overall rather small and, compared to this, sample sizes in the supplementary analysis were not overly large (*n*s = 459 and 346). Moreover, men and women differed with regard to practiced types of sport. In team sports, which dominated among men, unfamiliarity with, and negative perceptual frequency effects of, left-handed and left-sided movements of opponents (see Introduction) may be more important, and may thus lead to a better performance of persons with left preferences, than (small) gains in bodily coordination that might be a consequence of mixed preferences. Vice versa, in sports like swimming, that were more often practiced among women, effects of unfamiliarity and negative perceptual frequency regarding the movements of an opponent have no direct application; instead, better bodily coordination may result in better performance here. Hence, observed sex differences appear to be confounded by the different types of sport that were practiced by men and women in Study 2. Clearly, more research is needed here. In conclusion, the results of Study 2 broadly corroborated the findings of Study 1 by replicating positive effects of footedness on sporting performance, but refined them in pointing out that specifically movement-footedness, not skilled-footedness, predicts overall sporting performance in samples of everyday sportspersons.

## General Discussion

This series of studies investigated lateral preferences of hand and foot with regard to overall sporting performance and motor abilities in three large and independent general population samples. We obtained replicable evidence that footedness predicted sporting performance and motor abilities, observing that left-footedness and mixed-footedness granted benefits that are consistent with published findings on strategic advantages and negative frequency effects, but also with innate superior abilities of non-right-handers.

### Effects on Sporting Performance and Motor Abilities

Results corroborated an advantage of left preferences in team sports and martial arts and fencing that is most likely strategic or derives from negative frequency effects (see Faurie and Raymond, 2005; Hagemann, 2009; Loffing et al., 2012b; Wood and Aggleton, 1989). Footedness may have emerged as the more important predictor than handedness here, because it may be a more relevant indicator of cerebral lateralization than handedness (see below). Overall, lateral preferences for handedness and footedness were strongly concordant in our data; left-footers were mostly also left-handers. Specific effects of handedness (vs. footedness) with regards to sport types where the player holds a racket (e.g., tennis) or a weapon (i.e., fencing) could not be examined in our data, as respective sport types had only low prevalence rates. Specific effects of footedness still need to be investigated in these sport types in more detail in future research.

Study 2 provided evidence for sex differences of laterality effects for sporting performance and motor abilities. Previous research indicated that in tennis the advantage of left-handedness is smaller for women than for men (Breznik, 2013), and that visual reaction times are longer among female than male handball players, but equal – and shorter overall – among left-handed players of both sexes (Dane and Erzurumluoglu, 2003). We found that among men left-footedness, but among women mixed-footedness, exerted positive effects on sporting performance in Study 2. As these results were confounded by types of practiced sports, which differed between men and women, this finding may not be evidence of a true sex difference. More research is needed here.

Concerning motor abilities, we obtained evidence of laterality effects and sex differences thereof that might be hormone-related. Mixed- and left-footers (both sexes) reported higher flexibility, higher strength (men), and higher endurance (women), all of which appear to be consistent with theories of intrauterine testosterone-induced effects on cerebral lateralization (e.g., Geschwind and Galaburda, 1987), and effects of testosterone on fat mass, muscle strength, and leg power (Herbst and Bhasin, 2004), and on cardiovascular events among women (Sievers et al., 2010). There is also evidence of higher aggression among male left-handed soccer players (Dane and Sekertekin, 2005; see also Groothuis et al., 2014) which may or may not be testosterone-associated as well. More research on hormone-related effects in laterality research is currently needed.

Mixed-footedness appeared further indicative of higher coordination and speed, whose biological basis may stem from a better brain interhemispheric communication (Davidson and Tremblay, 2013). In the present series of studies, positive effects were not only observed in interactive sports, but also in non-interactive sports, where the performance depends on a good whole-body coordination and speed (i.e., dancing, swimming, and skiing). As previous research did not differentiate mixed from left preferences and focused only on handedness (e.g., Grouios et al., 2000), effects of mixed-footedness in non-interactive sports were obviously overlooked (but see e.g., Carey et al., 2001 and Grouios et al., 2002b, for interactive sports like soccer). Neurological studies may need to distinguish more clearly between the three classes of lateral preferences in future studies, using psychometrically sound scales and empirically obtained cutoffs (Tran et al., 2014a), to further elucidate the neural basis of such an effect. Studies also need to investigate footedness more closely.

Overall, effects of lateral preferences on sporting performance and motor abilities were only small in the present series of studies. However, it appears interesting to note that even though training may exert effects on sport-specific hand preference, these do not tend to generalize to everyday hand preference (Loffing et al., 2014; Stöckel and Vater, 2014; but see Maeda et al., 2014). Similarly, foot preference is also largely independent of, and not changeable through, training (Carey et al., 2001, 2009). Together with the fact that the present series of studies investigated primarily amateur and everyday sportspersons who did not partake in professional training, our results suggest that lateral preferences may have an effect on sporting ability and motor abilities, rather than sporting ability and training on lateral preferences. More research is needed here, though.

### Evolutionary Underpinnings

The so-called fighting hypothesis (FH; see Groothuis et al., 2014, for an overview and critical evaluation) posits that left-handedness may have evolved and persisted in human evolution, because of strategic advantages in fighting and frequency-dependent selection. However, previous evidence has been mixed and ambiguous with regard to interpretation (Groothuis et al., 2014). In the present series of studies, men were more likely to be left-footed and mixed-footed than women, and left-footedness was found specifically beneficial in martial arts and fencing. This pattern is consistent with the assumption that left preferences were important in a context of male–male competition (Faurie et al., 2011) and is in favor of the FH. However, the FH does currently not account for mixed preferences. Future research may need to consider mixed preferences more closely, whose effects on sporting performance and motor abilities may on the one hand relate to a better brain interhemispheric communication, but on the other hand also, like left preferences, on hormonal effects.

### Footedness

Our results are in line with findings suggesting that footedness may be a more relevant indicator of cerebral lateralization than handedness (Elias and Bryden, 1998; see also Tran et al., 2014a). Previous studies provided evidence of the possible multidimensionality of self-reported footedness (e.g., Kalaycıoğlu et al., 2008; Schneiders et al., 2010), but suffered from methodological weaknesses. In contrast, the present study utilized appropriate statistical methods, showing that footedness is a two-dimensional construct. This multidimensionality of footedness needs to be considered in laterality research and with regard to questions of assessment (Tran et al., 2014a). The 8-item scale utilized here is recommended for future studies.

Footedness is considered phylogenetically primary to handedness in evolutionary ‘postural control’ theories (e.g., Day and MacNeilage, 1996), suggesting that postural demands of unimanual predation led to a right-side, left-brain hemisphere specialization. Previous studies failed to provide evidence of movement-footedness being specifically predictive of cerebral lateralization (Elias et al., 1998). However, previous research did not utilize items such as ‘stepping forward’ or ‘stepping up stairs,’ which proved the best indicators of movement-footedness in Study 2. Hence, more research is still needed here.

### Limitations

The utilized measure of sporting performance did not allow a detailed and direct assessment of sporting ability and success. More detailed instruments and direct measurements (e.g., of time or speed), matching specific sport-related abilities, are needed in future studies. Further, only self-reported lateral preferences were investigated. Measures of ability or performance may also need to be examined in future research. The present research did not select participants with regard to their practiced type of sport or their sporting performance. Future investigations should thus examine specific sport types and selected samples of professional athletes.

## Conclusion

The present series of studies obtained replicable evidence of footedness being a more relevant predictor of sporting performance and motor abilities than handedness. Specifically mixed- and left-footedness showed positive effects in various interactive and non-interactive sports, suggesting better bodily coordination and speed, but also strategic advantages that are consistent with frequency-dependent effects. Laterality effects were only small and observed sex differences are in need of further study. However, laterality effects appear interesting with regard to evolutionary theories of handedness and lateral preferences. Possible neural and hormonal bases of observed effects need to be examined in future studies.

## Author Contributions

UT contributed to the conception and design of the study, conducted the research, conducted the analyses, and drafted the manuscript. MV had the original idea for the study and contributed in drafting the manuscript.

## Acknowledgment

This article was supported by the Open Access Publishing Fund of the University of Vienna.

## Conflict of Interest Statement

The authors declare that the research was conducted in the absence of any commercial or financial relationships that could be construed as a potential conflict of interest.

## Footnotes

1 Scored 1 = I do no sport, 2 = My sport is only social, 3 = I think I could compete at an organized level, 4 = I have competed at an organized level, 5 = I think I could compete at county level, 6 = I have competed at county level, 7 = I think I could compete at national level, 8 = I have competed at national level, 9 = I think I could represent my country, 10 = I have represented my country.
